# Profile of leisure-time physical activity and sedentary behavior in adults in Brazil: a nationwide survey, 2019

**DOI:** 10.1590/S2237-96222023000200016

**Published:** 2023-08-11

**Authors:** Arão Belitardo de Oliveira, Peter T. Katzmarzyk, Wagner Silva Dantas, Isabela Judith Martins Benseñor, Alessandra de Carvalho Goulart, Ulf Ekelund

**Affiliations:** 1 Centro de Pesquisa Clínica e Epidemiológica, Hospital Universitário, Universidade de São Paulo, São Paulo, Brazil; 2 Pennington Biomedical Research Center, Louisiana State University, Baton Rouge, Estados Unidos da América; 3 Department of Sport Medicine, Norwegian School of Sport Sciences, Oslo, Noruega

**Keywords:** Physical Activity, Aerobic Exercise, Physical Fitness, Strength Training, Sedentary Behavior, Actividad Física, Ejercicio Aeróbico, Condición Física, Entrenamiento de Fuerza, Sedentarismo, Atividade Física, Exercício Aeróbico, Aptidão Física, Treinamento de Força, Comportamento Sedentário

## Abstract

**Objectives:**

to estimate the prevalence of leisure-time physical activity and sedentary behavior in adults in Brazil.

**Methods:**

this was a cross-sectional, population-based study carried out in a sample of 88,531 Brazilians, using data from the 2019 National Health Survey; leisure-time physical activity (overall and aerobic exercise) was measured according to the World Health Organization guidelines; the weighted prevalence and respective 95% confidence intervals (95%CI) of physical activity, physical inactivity and sedentary behavior were estimated.

**Results:**

according to the selected sample, 26.4% (95%CI 25.9;27.1) of Brazilian adults were physically active, 14.0% (95%CI 13.5;14.4) were insufficiently physically active and 59.5% (95%CI 58.8;60.2) were physically inactive; sedentary behavior ≥ 6 hours was reported by 30.1% (95%CI 29.5;30.8) of the population; only 8.6% (95%CI 8.2;8.9) met the recommendations for muscle-strengthening activities.

**Conclusion:**

most Brazilian adults were physically inactive and did not meet international recommendations for leisure-time physical activity and reduction in sedentary behavior.

## Resumo

**Objetivos:**

estimar a prevalência de atividade física no tempo livre e comportamento sedentário em adultos no Brasil.

**Métodos:**

estudo seccional, populacional, na amostra de 88.531 brasileiros da Pesquisa Nacional de Saúde de 2019; a atividade física (geral e modalidades aeróbias) no tempo livre foi mensurada conforme as diretrizes da Organização Mundial da Saúde; estimaram-se as prevalências ponderadas, e respectivos intervalos de confiança de 95% (IC_95%_), de atividade física, inatividade física e tempo sedentário.

**Resultados:**

a partir da amostra selecionada, 26,4% (IC_95%_ 25,9;27,1) dos adultos brasileiros eram fisicamente ativos, 14,0% (IC_95%_ 13,5;14,4) eram insuficientemente ativos e 59,5% (IC_95%_ 58,8;60,2) eram inativos; o tempo sedentário ≥ 6 horas foi relatado por 30,1% (IC_95%_ 29,5;30,8) da população; apenas 8,6% (IC_95%_ 8,2;8,9) atenderam às recomendações de atividade física para fortalecimento muscular.

**Conclusão:**

a maioria dos brasileiros adultos era inativa e não atendeu às recomendações internacionais de atividade física no tempo livre e restrição de tempo sedentário.

## Resumen

**Objetivos:**

estimar la prevalencia de actividad física en el tiempo libre y comportamiento sedentario de adultos en Brasil.

**Métodos:**

estudio seccional, poblacional, de la muestra de la Encuesta Nacional de Salud de 2019 a partir de lo cual, se estimó la prevalencia ponderada (porcentaje e intervalo de confianza del 95%) de actividad física (general y aeróbica), la inactividad física y el comportamiento sedentario.

**Resultados:**

según la muestra elegida, 26,4% (IC_95%_ 25,9;27,1) son físicamente activos, 59,5% (IC_95%_ 58,8;60,2) son inactivos, 14,0% (IC_95%_ 13,5;14,4) son insuficientemente activos y 30,1% (IC_95%_ 29,5;30,8) pasan ≥6 horas sedentarios en su tiempo libre; sólo el 8,6% (IC_95%_ 8,2;8,9) de los adultos cumple con las recomendaciones de actividad física para fortalecimiento muscular.

**Conclusión:**

la mayoría de los adultos brasileños son inactivos, no cumplen con las recomendaciones internacionales de actividad física en el tiempo libre y la restricción del tiempo sedentario.

**Table:** 

Study contributions	
**Main results**	The majority of Brazilian adults were physically inactive, with 26.4% meeting the recommendations for leisure-time physical activity. Only 8.6% met the recommendations for muscle-strengthening activities, and approximately one-third of them showed a sedentary behavior during most of their leisure time.
**Implications for services**	The data will help guide and update health promotion programs at the Primary Healthcare Center (PHC) that include the recommendations for physical activity, with a focus on leisure-time physical activity.
**Perspectives**	Further studies are needed to assess whether surveillance of levels of leisure-time physical activity and sedentary behavior in the Brazilian population can positively impact on the redesign of health promotion programs at PHCs.

## INTRODUCTION

The World Health Organization (WHO), in its latest guidelines, recommends moderate-intensity (≥ 150 minutes) or vigorous-intensity (≥ 75 minutes) aerobic physical activity a week, respectively. Adults should also do muscle-strengthening activities two days a week, aiming at reducing risk of chronic diseases. In addition, it has recommended the reduction in sedentary behavior.^11^

Population-based data on surveillance of leisure-time physical activity in Brazil require constant updates. The analyses of the most recent data have not explored the prevalence of leisure-time physical activity by aerobic physical activity and muscle strengthening modalities.^2^ ^)-(^ ^5^ This analysis allows us to assess the levels of specific physical activity with reference to international recommendations. The objective of this study was to analyze data from the 2019 National Health Survey (*Pesquisa Nacional de Saúde* - PNS 2019), conducted by the Brazilian Ministry of Health.^3^ The levels of leisure-time physical activity and sedentary behavior in Brazilian adults were estimated according to recent WHO recommendations.

## METHODS

*Study design*

This was a cross-sectional, population-based study among the participants of the PNS 2019.

*Setting*

The study comprised a cross-sectional analysis of data from the PNS 2019, a household survey conducted nationwide, both in urban and rural areas, composing a representative sample of the Brazilian population. The PNS 2019 used the three-stage conglomerate plan, with primary sampling unit stratification. These units consisted of census tracts or sets of tracts, and the selection was obtained by means of simple random sampling among those previously selected for the study.^6^ The expected coefficient of variation (CV) was calculated based on values of indicators estimated from previous data obtained from the PNS 2013, using formulas for a sampling plan by simple random sampling and the effect of the sampling plan also estimated by the PNS 2013.^6^ The CV was adjusted because the conglomerate plan is less efficient than the simple random sampling. In addition, the sample sizes of households and individuals were adjusted to fit the master sample, which serves as the sampling infrastructure for the research. The number of occupied households according to the Population Census was used as a measurement of the census tract size. The PNS 2019 was conducted by the Brazilian Institute of Geography and Statistics (*Instituto Brasileiro de Geografia e Estatística* - IBGE), in partnership with the Ministry of Health, between August 2019 and March 2020.^6^

A total of 94,111 households were randomly selected and 90,846 household residents, aged ≥ 15 years, were interviewed (response rate of 93.6%), and answered the questions in Module P contained in the 2019 PNS questionnaire regarding lifestyle information.

*Participants*

**Figure 1:**
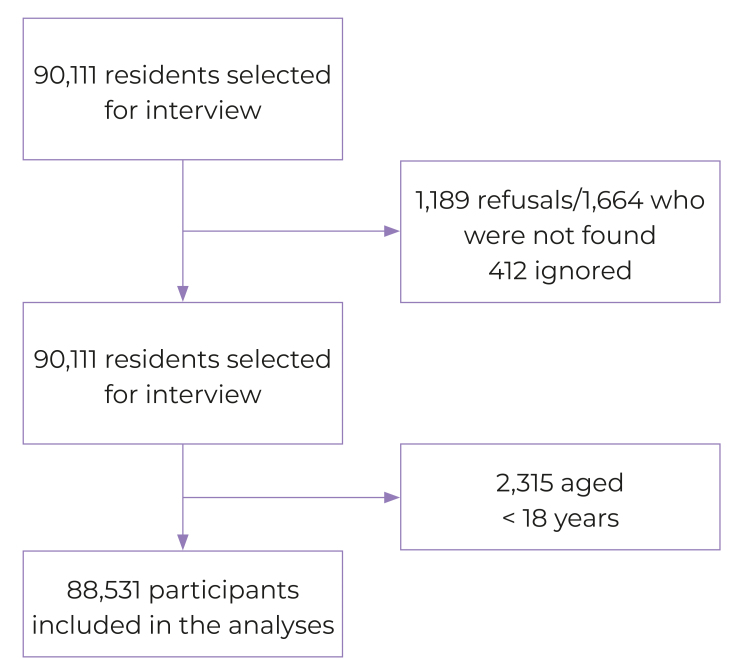
Process of inclusion of participants in the survey (n = 88,531)

Participants aged ≥ 18 years were included in this analysis, with complete information about age, sex, leisure-time physical activity and sedentary behavior. Figure 1 shows the population flow included in the analysis of this study.

*Variables*

The study variables were: leisure-time physical activity (physically inactive, insufficiently physically active and physically active), both for aerobic physical activity and muscle-strengthening modalities, and sedentary behavior (in hours, categorized as 0 to < 4, 4 to < 6, 6 to < 8 and ≥ 8 hours).

Participation in any leisure-time physical activity in the last three months (yes/no), typical weekly frequency (0-7 days), duration (minutes or hours a day) and exercise modality performed (among 16 options) were self-reported.

The total weekly leisure-time physical activities, in minutes, was calculated and categorized according to the 2020 WHO physical activity guidelines, using the minimum threshold of 150 minutes/week for total leisure-time physical activity or moderate- to vigorous-intensity aerobic physical activity.^1^ Participants who reported doing muscle-strengthening activities two or more times a week were categorized as physically active for this modality, as established by WHO guidelines.^1^

The response options for the modalities performed for categorizing physical activity are outlined below.

- Aerobic activities: “walking”, “treadmill walking”, “jogging or running”, “running on treadmill”, “aerobic gymnastics/spinning/step/jumping”, “water aerobics”, “swimming”, “martial arts and wrestling”, “cycling or stationary bike”, “football”, “basketball”, “volleyball”, “tennis”, “dance class”.

-Muscle-strengthening activities: “weight training” and “localized gymnastics/Pilates/stretching or yoga”.

Daily screen time was measured as the self-reported time spent watching TV and using computer/laptop/tablet/cell phone during leisure time, and was operationalized as sedentary behavior.^7^ *Daily screen time was estimated by aggregating the responses to two questions:* (i) *On average, how many hours per day do you spend watching TV?;* and (ii) *How many hours of your free time per day (excluding work) do you usually spend using a computer, tablet or cell phone as a form of leisure, such as using social networks, watching news, videos, playing games, etc.?*

Sex assigned at birth (male; female) and age in years (categorized into 18-34, 35-49, 50-64, and ≥ 65) were selected as covariates.

*Data sources and measurement*

The 2019 PNS data are publicly available and the microdata were downloaded from the website https://www.ibge.gov.br/estatisticas/sociais/saude/9160-pesquisa-nacional-de-saude.html?=&t=microdados. The data were analyzed between February and April 2022.

*Bias control*

All participants who responded to the survey and met the inclusion criterion, age ≥ 18 years, answered the questions related to the variables of interest, therefore, there was no selection bias.

*Statistical methods*

Data were analyzed using the Stata statistical software (version 17.0, StataCorp LLC) and svy commands for analysis of complex samples, with weights for non-response adjustments; post-stratification adjustments were applied. The weighted prevalence estimates, described as percentage (%) and 95% confidence interval (CI), were stratified by sex and age group.

*Ethical aspects*

The study was approved by the National Research Ethics Committee on December 23, 2019, No. 3,529,376. Participants provided written informed consent prior to the interview.

## RESULTS

The flow of participants included in this study is shown in Figure 1. All participants aged ≥ 18 years answered the questions related to the variables of interest (n = 88,531).

In the total sample size, 22,253 Brazilian adults (26.4%; 95%CI 25.9;27.1) were physically active, 11,486 (14.0%; 95%CI 13.5;14.4) were insufficiently physically active and 54,792 (59.5%; 95%CI 58.8;60.2) were physically inactive in their leisure time. Sedentary behavior ≥ 6 hours was reported by 27,821 (30.1%; 95%CI 29.5;30.8) of Brazilian adults.

Among the participants who reported having performed any physical activity in the previous three months (n = 33,739), the weighted prevalence of the three most frequent physical activity modalities were as follows: walking (36.0%; 95%CI 35.1;36.9), resistance training (17.5%; 95%CI 16.8;18.3) and soccer (16.6%; 95%CI 15.9;17.9).

Female people showed higher prevalence of physical inactivity (63.1%; 95%CI 62.2; 63.9) when compared to male participants (55.5%; 95%CI 54.5;56.4). There was a consistent pattern of lower prevalence of leisure-time physical activity with increasing age (Table 1).

**Table 1:** Prevalence of leisure-time physical activity by self-reported sedentary behavior, sex and age group, in 88,531 adults in Brazil, 2019

Variables	Overall		Sedentary behavior (hours)							
			0;4		4;6		6;8		≥ 8	
	N	95%CI	n	95%CI	n	95%CI	n	95%CI	n	% (CI)
Male (41,662)										
Inactives	24,808	55.5 (54.5;56.4)	13,171	25.8 (25.0;26.5)	6,338	15.6 (15.0;16.2)	3,598	9.3 (8.8;9.9)	1,701	4.7 (4.3;5.2)
Insufficiently active	6,177	16.2 (15.6;16.9)	2,432	5.7 (5.3;6.1)	2,029	5.5 (5.1;5.9)	1,130	3.2 (2.9;3.6)	586	1.7 (1.5;2.5)
Active	10,677	28.3 (27.5;29.2)	3,551	8.8 (8.3;9.3)	3,581	9.0 (8.5;9.5)	2,311	6.6 (6.1;7.1)	1,234	3.8 (3.4;4.2)
Active by modality										
Aerobic	8,373	21.9 (21.1;22.7)	2,986	7.3 (6.8;7.7)	2,743	6.7 (6.3;7.2)	1,726	5.0 (4.6;5.4)	918	2.7 (2.4;3.1)
Muscle-strengthening	2,611	7.2 (6.7;7.7)	690	1.8 (1.6;2.1)	932	2.4 (2.2;2.7)	643	1.7 (1.5;2.0)	346	1.1 (0.9;1.4)
Female (46.869)										
Inactive	29,984	63.1 (62.2;63.9)	12,974	24.6 (23.8;25.3)	8,444	19.0 (18.3;19.6)	5,676	12.6 (12.1;13.1)	2,890	6.9 (6.5;7.3)
Insufficiently active	5,309	12.1 (11.5;12.6)	2,167	4.7 (4.4;5.1)	1,767	4.3 (3.9;4.7)	945	1.9 (1.7;2.2)	430	1.0 (0.8;1.2)
Active	11,576	24.9 (24.1;25.6)	3,993	8.3 (7.9;8.7)	3,896	8.4 (7.9;8.8)	2,411	5.2 (4.9;5.6)	1,276	2.8 (2.5;3.1)
Active by modality										
Aerobic	8,181	17.2 (16.6;17.9)	3,093	6.2 (5.8;6.5)	2,690	5.8 (5.4;6.2)	1,590	3.4 (3.1;3.7)	808	1.7 (1.5;2.0)
Muscle-strengthening	4,072	9.0 (8.6;9.5)	1,135	2.6 (2.3;2.8)	1,461	3.1 (2.8;3.4)	951	2.1 (1.9;2.3)	525	1.2 (1.0;1.4)
Age (anos) 18-29 (n = 24.115)										
Inactive	12,689	51.2 (50.0;52.4)	3,724	12.3 (11.7;13.0)	3,637	15.1 (14.2;16.0)	3,171	13.8 (13.0;14.6)	2,157	10.0 (9.3;10.8)
Insufficiently active	3,731	16.1 (15.3;17.0)	1,053	3.9 (3.4;4.4)	1,232	5.4 (4.9;5.9)	884	4.0 (3.6;4.5)	562	2.6 (2.3;3.0)
Active	7,695	32.7 (31.5;33.8)	1,781	7.0 (6.4;7.6)	2,488	10.1 (9.4;10.9)	2,102	9.3 (8.6;10.1)	1,324	6.1 (5.5;6.7)
Active by modality										
Aerobic	5,095	21.3 (20.3;22.3)	1,274	4.5 (4.1;5.0)	1,593	6.4 (5.8;6.9)	1,357	6.2 (5.6;6.8)	871	4.0 (3.6;4.6)
Muscle-strengthening	2,857	12.6 (11.9;13.4)	568	2.7 (2.3;3.1)	988	4.1 (3.7;4.6)	810	3.4 (3.0;3.9)	491	2.2 (1.9;2.5)
30-45 (n = 26.031)				
Inactive	15,589	58.8 (57.6;60.1)	7,040	24.4 (23.4;25.3)	4,639	19.0 (18.1;20.0)	2,577	10.2 (9.5;10.8)	1,333	5.2 (4.8;5.7)
Insufficiently active	3,530	14.3 (13.5;15.1)	1,461	5.6 (5.1;6.1)	1,210	5.3 (4.7;6.0)	610	2.3 (2.1;2.6)	249	0.9 (0.7;1.1)
Active	6,912	26.9 (25.9;27.8)	2,411	9.3 (8.7;9.9)	2,378	9.1 (8.6;9.8)	1409	5.5 (5.0;6.0)	714	2.8 (2.2;3.5)
Active by modality										
Aerobic	5,019	19.6 (18.7;0.4)	1,842	7.2 (6.7;7.7)	1,702	6.6 (6.1;7.2)	984	3.8 (3.4;4.2)	491	1.8 (1.6;2.2)
Muscle-strengthening	2,142	8.2 (7.6;8.8)	665	2.5 (2.1;2.8)	762	2.8 (2.5;3.1)	470	1.8 (1.6;2.1)	245	1.0 (0.7;1.4)
46-64 (n = 22.459)										
Inactive	14,820	63.4 (62.2;64.7)	8,180	32.8 (31.7;33.9)	3,811	17.9 (17.0;18.8)	2,025	9.0 (8.4;9.6)	804	3.7 (3.3;4.1)
Insufficiently active	2,585	13.0 (12.2;13.9)	1,214	6.1 (5.5;6.7)	869	4.5 (4.0;5.1)	360	1.6 (1.3;1.9)	142	0.6 (0.5;0.8)
Active	5,054	23.6 (22.5;24.7)	2,069	9.7 (9.0;10.5)	1,762	8.1 (7.5;8.8)	854	3.9 (3.4;4.4)	369	1.6 (1.4;2.0)
Active by modality										
Aerobic	4,214	19.7 (18.7;20.7)	1,805	8.4 (7.7;9.0)	1,435	6.7 (6.2;7.3)	689	3.2 (2.8;3.7)	285	1.2 (1.0;1.5)
Muscle-strengthening	1,097	5.1 (4.6;5.6)	362	1.8 (1.5;2.1)	428	1.9 (1.6;2.3)	210	0.8 (0.6;0.9)	97	0.5 (.03;0.7)
≥ 65 (n = 15.926)										
Inactive	11,694	72.3 (71.1;73.5)	7,201	41.8 (40.0;43.1)	2,695	18.3 (17.4;19.4)	1,501	10.2 (9.4;11.1)	297	2.0 (1.6;2.4)
Insufficiently active	1,640	10.6 (9.7;11.4)	871	5.5 (5.0;6.1)	485	3.1 (2.7;3.7)	221	1.2 (1.0;1.5)	63	0.5 (0.3;0.7)
Active	2,592	17.1 (16.1;18.2)	1,283	8.4 (7.7;9.2)	849	5.4 (4.8;6.0)	357	2.3 (1.9;2.7)	103	0.8 (0.6;1.2)
Active by modality										
Aerobic	2,226	14.8 (13.8;15.8)	1,158	7.6 (6.9;8.4)	703	4.5 (4.0;5.1)	286	1.8 (1.5;2.2)	79	0.6 (0.4;0.9)
Muscle-strengthening	587	3.7 (3.3;4.2)	230	1.3 (1.1;1.6)	215	1.3 (1.1;1.7)	104	0.6 (0.4;0.8)	38	0.3 (0.2;0.6)

**Figure 2:**
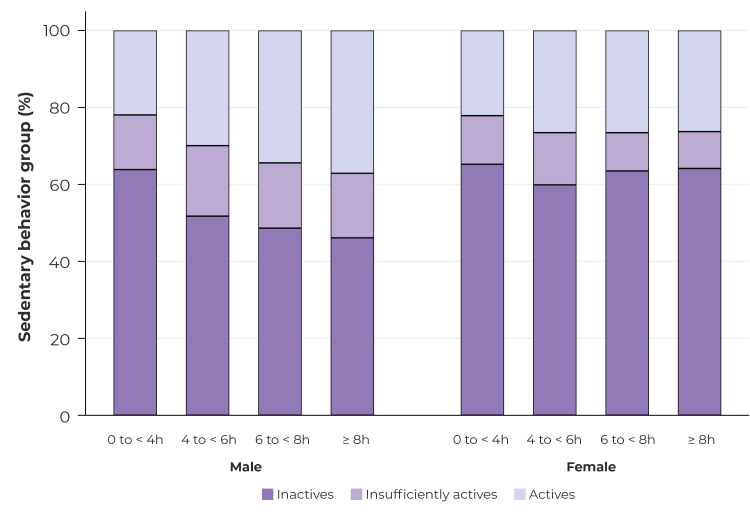
The prevalence of self-reported leisure-time physical activity by sedentary behavior groups and sex, in the PNS 2019, Brazil (n = 88,531)

Only 19.4% (95%CI 18.9;19.9) of Brazilian adults met the leisure-time physical activity recommendations for moderate- to vigorous-intensity aerobic physical activity modalities, while only 8.6% (95%CI 8.2;8.9) met the recommendations for the muscle-strengthening modality. The prevalence of physically active Brazilian adults by age group, sex and sedentary behavior groups is summarized in Table 1. Male participants showed higher levels of leisure-time physical activity in aerobic exercise modalities, when compared to female participants, while female participants showed higher levels of leisure-time physical activity in muscle-strengthening modality (Table 1). However, the proportion of physically active male increased in the groups with higher sedentary behavior, while among female, physical inactivity was higher than among male in all sedentary behavior groups (Figure 2).

## DISCUSSION

This study showed a low prevalence of leisure-time physical activity and a high prevalence of sedentary behavior in the Brazilian adult population. In the United States, a study indicated that 40.4% of the adult population met the WHO recommendations for leisure-time physical activity, while 44.6% remained inactive.^8^ In that country, measures to address physical inactivity and sedentary behavior focused on primary care interventions.^9^ ^)-(^ ^11^ Comparisons with other countries may be limited due to variation in methodologies for estimating physical activity. In the present study, only the leisure time domain was analyzed, whereas other countries such as England and China have analyzed other domains of physical activity, including transportation and occupational activities.^12^ ^),(^ ^13^ In this context, a 2018 estimate on global physical inactivity, based on previous population studies, showed that Brazil still had one of the highest levels of physical inactivity in Latin America.^14^

Regarding the prevalence of muscle-strengthening activities, data from the PNS 2019 revealed a worrying prevalence. Studies suggest that this modality of physical activity can also prevent metabolic and cardiovascular diseases in adults^15^ ^),(^ ^16^ and reduce mortality.^17^ ^)-(^ ^19^ It is crucial that health policy actions prioritize muscle-strengthening modalities.

As for sedentary behavior, the percentage of people who meet the WHO recommendations for leisure-time physical activity increased as more time was spent in sedentary behavior among males, but not among females. Sociodemographic and occupational factors may have determined differences between sexes, favoring more time for physical activity among males. Data from prospective studies have indicated that increasing levels of leisure-time physical activity may counterbalance the detrimental effects of sedentary behavior on health.^7^ ^),(^ ^17^ ^),(^ ^19^ This finding should be considered by police-makers as an opportunity to develop sex-specific interventions aimed at reducing sedentary behavior.

Several public health actions and interventions to promote physical activity have been implemented in primary health care, with examples showing positive results in levels of leisure-time physical activity in some places in Brazil.^20^ ^)-(^ ^24^ In a systematic review on the effect of counseling and guidance interventions for physical activity in primary health care, the results ranged from no change to an increase of 88 minutes per week in the initial levels of participants’ leisure-time physical activity.^24^ However, a 2014 national study, which randomly selected 1,600 primary health care centers (PHC), showed that only 39.8% had physical activity promotion program.^25^ These findings, combined with our data, indicate that prevention programs in primary health care need to be updated in their priorities and expanded, with a focus on leisure-time physical activity.

The limitations of this study are related to self-reported data on leisure-time physical activity, the lack of data on the time individuals spend sitting in the workplace and the data that have not been validated for sedentary time yet. The strength of this study was the use of the PNS 2019, which is the largest and most comprehensive face-to-face health survey in Brazil, which included a representative sample of the Brazilian population and recruited highly qualified and trained staff for data collection and processing.

In summary, the majority of the Brazilian population demonstrated physical inactivity and sedentary behavior in their leisure time. This study provides evidence for policymakers and healthcare professionals responsible for primary care programs, such as PHCs, to prioritize specific populations and modalities of leisure-time physical activity.
